# D^2^MNet: Difference-Aware Decoupling and Multi-Prompt Learning for Medical Difference Visual Question Answering

**DOI:** 10.3390/jimaging12040162

**Published:** 2026-04-09

**Authors:** Lingge Lai, Weihua Ou, Jianping Gou, Zhonghua Liu

**Affiliations:** 1 School of Big Data and Computer Science, Guizhou Normal University, Guiyang 550025, China; lailingge@gznu.edu.cn; 2 School of Computer and Information Sciences, Southwest University, Chongqing 400715, China; cherish.gjp@gmail.com; 3 School of Information Engineering, Zhejiang Ocean University, Zhoushan 316022, China; lzhua_217@163.com

**Keywords:** medical vision question answering, chest X-ray, multi-prompt learning

## Abstract

Difference visual question answering (Diff-VQA) aims to answer questions by identifying and reasoning about differences between medical images. Existing methods often rely on simple feature subtraction or fusion to model image differences, while overlooking the asymmetric descriptive requirements of changed and unchanged cases and providing limited task-specific guidance to pretrained language decoders. To address these limitations, we propose D^2^MNet (Difference-aware Decoupling and Multi-prompt Network), a framework for medical Diff-VQA that combines change-aware reasoning with prompt-guided answer generation. Specifically, a Change Analysis Module (CAM) predicts whether a change is present and produces a binary change-aware prompt; a Difference-Aware Module (DAM) uses dual attention to capture fine-grained difference features; and a multi-prompt learning mechanism (MLM) injects question-aware, change-aware, and learnable prompts into the language decoder to improve contextual alignment and response generation. Experiments on the MIMIC-DiffVQA benchmark show that D2MNet achieves a CIDEr score of 2.907 ± 0.040, outperforming the strongest baseline, ReAl (2.409), under the same evaluation setting. These results demonstrate the effectiveness of the proposed design on benchmark medical Diff-VQA and suggest its potential for assisting difference-aware medical answer generation.

## 1. Introduction

Visual question answering (VQA) has emerged as a rapidly evolving research paradigm that integrates computer vision and natural language processing to answer natural language questions based on visual content [1,2]. In the field of general-purpose imagery, VQA models have demonstrated strong capabilities in extracting and reasoning over complex scene elements, showing promise for applications such as education, robotics, and assistive technologies. Building on this foundation, researchers have extended VQA to the medical imaging domain, leading to the development of medical visual question answering (Med-VQA). This task aims to interpret medical images and generate medically meaningful answers to diagnostic questions, thereby providing intelligent assistance for medical image understanding and clinical workflow support [3,4,5,6].

With the growing demand for medical image interpretation, the field is facing critical challenges, including overwhelming workloads for radiologists and a shortage of trained medical professionals [7]. In clinical practice, radiologists are often required to compare a patient’s current imaging studies with historical scans to assess disease progression and evaluate treatment response. However, such comparative analysis requires substantial expertise and sustained attention, further exacerbating the burden on physicians [8,9,10]. Despite recent progress, most existing Med-VQA methods remain constrained to single-image analysis and lack the capability to interpret complex clinical scenarios involving multi-time point comparisons. As a result, they are not well suited to the practical need for reasoning about differences between past and present images.

To address this limitation, researchers have begun exploring Med-VQA methodologies tailored to multi-image difference scenarios. Hu et al. [11] first introduced the difference visual question answering (Diff-VQA) task together with the MIMIC-Diff-VQA dataset and proposed the Expert Knowledge-Aware Image Difference (EKAID) model, which uses expert knowledge-based graph representation learning to capture change information between medical images for answer generation. Cho et al. [12] proposed a pretrained vision–language model, PLURAL, which is first pretrained on natural images and texts and then fine-tuned on longitudinal chest radiographs to improve the understanding and description of temporal changes in medical imaging. In addition, Lu et al. [13] proposed the Residual Alignment (ReAl) model, which introduces a dedicated residual encoding branch to capture subtle differences between medical images.

### 1.1. Literature Gaps

However, existing Diff-VQA approaches still face several important challenges. First, they often overlook the asymmetric generation requirements of changed and unchanged scenarios. When no meaningful difference exists, a concise and standardized answer may be sufficient, whereas cases with pathological progression require more detailed and fine-grained descriptions. Treating both scenarios in a uniform manner may therefore lead to outputs that are either unnecessarily verbose or insufficiently informative. Second, many methods rely on simple strategies, such as feature subtraction or naive concatenation, to model image differences. Such operations may fail to preserve subtle yet important variations that are essential for accurate reasoning. Third, although pretrained language models have shown strong generation ability, existing approaches rarely incorporate task-specific guidance into the decoding process, which limits the generation of answers that are both contextually appropriate and structurally consistent. These limitations reduce the precision and consistency of generated responses in medical Diff-VQA. These gaps motivate the need for a framework that can explicitly distinguish change scenarios, capture fine-grained differences, and guide answer generation in a task-specific manner.

### 1.2. Our Contributions

To address these challenges, we propose a novel architecture named D^2^MNet (Difference-aware Decoupling and Multi-prompt Network), specifically designed for medical difference visual question answering. First, a Change Analysis Module (CAM) is designed to explicitly predict whether a meaningful change exists between the image pair, enabling the model to distinguish different scenario types and adjust its response behavior accordingly. Second, a Difference-Aware Module (DAM) incorporates dual attention mechanisms to capture fine-grained variations, improving the extraction of subtle yet critical difference features. Third, a multi-prompt learning mechanism (MLM) integrates question-aware, change-aware, and learnable prompts into the pretrained language decoder, providing task-specific guidance that steers the generation process toward accurate and structurally consistent answer generation. Together, these modules form a unified framework that connects visual difference understanding with answer generation in medical Diff-VQA.

The main contributions of this paper are as follows:

- We propose D^2^MNet, a task-specific framework for medical Diff-VQA that combines change analysis, difference-aware feature modeling, and multi-prompt conditioned answer generation.
- We design a Change Analysis Module (CAM) and a Difference-Aware Module (DAM) to distinguish different change scenarios and capture subtle spatial–temporal difference features for more effective reasoning.
- We introduce a multi-prompt learning mechanism (MLM) that integrates question-aware, change-aware, and learnable prompts into the decoder to improve contextual alignment and answer generation.

## 2. Related Works

### 2.1. Difference Medical Visual Question Answering

Difference visual question answering (Diff-VQA) is an emerging task in medical imaging analysis, derived from recent advancements in medical visual question answering (Med-VQA) [11]. Unlike conventional Med-VQA methods that primarily focus on answering clinical questions based on single medical images, Diff-VQA considers pairs of longitudinal images from the same patient to answer questions related to disease progression, treatment response, and other difference-focused medical findings. This task is motivated by the longitudinal comparison process commonly performed in clinical practice, in which radiologists typically compare current medical examinations with historical imaging studies to assess disease evolution or treatment efficacy. For instance, in lung nodule follow-up scenarios, radiologists often compare CT images from different time points to ascertain whether a nodule has grown (potential indication of malignancy) or whether pulmonary lesions have improved post-treatment [14]. Therefore, Diff-VQA provides a useful research setting for studying difference-aware reasoning and answer generation in medical imaging.

Despite recent progress, one of the central challenges in Diff-VQA is how to accurately capture and model subtle differences between longitudinal medical images. Such differences may appear in fine-grained spatial or temporal patterns and can be easily affected by imaging noise, acquisition inconsistencies, or anatomical variability. To address this issue, several representative methods have been proposed. Hu et al.’s EKAID model [11] introduces expert knowledge-aware graph representations to model change information between medical images for answer generation. Cho et al.’s PLURAL [12] adopts large-scale pretraining on longitudinal chest X-ray data to improve the understanding and description of temporal changes in medical imaging. Lu et al.’s ReAl [13] incorporates a residual input branch and a residual feature alignment module to enhance sensitivity to subtle changes. In addition, simpler techniques, such as feature subtraction or concatenation [15,16], offer straightforward ways to model image differences but may have limited capacity to represent nuanced variations. Attention-based and graph-structured approaches further improve the modeling of relevant regions, yet many existing methods still treat changed and unchanged scenarios in a similar manner.

Although these studies have advanced Diff-VQA, several challenges remain. First, subtle pathological changes, together with imaging noise and positional inconsistency, make reliable difference modeling difficult. Second, existing methods often process all input scenarios in a uniform way, without explicitly considering the different generation requirements of changed and unchanged cases. Third, while pretrained language models provide strong generative ability, many approaches still lack task-specific guidance for answer generation. These issues continue to limit the precision and consistency of generated responses in medical Diff-VQA.

More recently, foundation-model-based directions have also emerged in medical vision–language research. For example, CheXagent introduces a chest X-ray vision–language foundation model trained on a large-scale instruction-tuning dataset [17], while CXR-LLaVA develops an open-source multimodal large language model for chest X-ray interpretation [18]. More broadly, RadFM explores a generalist radiology foundation model using web-scale 2D and 3D medical data [19]. These recent studies highlight the growing importance of stronger general-purpose medical vision–language backbones. However, they do not directly resolve the task-specific challenges of medical Diff-VQA, such as paired longitudinal reasoning, distinguishing changed from unchanged scenarios, and generating concise but clinically meaningful difference-focused answers. In this sense, our work is complementary to these recent trends, with a stronger emphasis on structured change-aware reasoning and controllable answer generation.

### 2.2. Multi-Prompt Learning

Prompt learning was first introduced in natural language processing (NLP) to guide pretrained language models toward specific tasks through task-related prompts, thereby reducing the need for extensive task-specific fine-tuning [20]. For example, large-scale language models such as GPT-3 [21] have demonstrated remarkable zero-shot and few-shot learning capabilities when provided with appropriate task descriptions or illustrative examples as prompts. This concept has subsequently been extended to vision–language modeling (VLM), where textual or visual prompts are integrated into joint image–text models to enhance downstream task performance [22,23,24]. In tasks such as visual question answering and image captioning, prompt-based learning helps models focus on task-relevant information [25].

In the medical domain, however, medical visual question answering (Med-VQA) faces challenges such as semantic misalignment between medical images and natural language, complex clinical questions, and the requirement of specialized medical knowledge [26,27]. Medical imaging encompasses diverse modalities and anatomical structures, and the associated questions often involve professional terminology, demanding precise visual comprehension and extensive domain knowledge. To address these challenges, researchers have begun incorporating prompt learning strategies into Med-VQA to bridge the semantic gap between visual and linguistic modalities [28]. For instance, some approaches map medical image features into learnable prompt embeddings and feed them into large-scale language models to generate open-ended responses. Van Sonsbeek et al. proposed a method that maps extracted visual features into a set of learnable prefix tokens, which are then used to prompt the language model together with the question, enabling open-ended medical visual question answering [29]. Other methods construct textual prompts from medical reports or clinical records to further align image representations with textual questions and guide the model toward critical diagnostic information. Chen et al. introduced a multi-question learning (MMQL) approach that uses previously answered questions as prompts to improve the diagnosis and response to related medical queries [28].

Nevertheless, current prompt learning methods in Med-VQA have limitations [30,31]. Many existing methods rely heavily on rigid templates or fixed prompt formats, applying identical prompt strategies across different question types. Such designs provide limited flexibility for adapting the model to the specific requirements of different generation scenarios, such as binary versus open-ended questions or changed versus unchanged cases [32,33]. As a result, these methods may not fully exploit task-dependent contextual cues and may show limited robustness when generalizing to diverse question settings. Therefore, developing adaptive multi-prompt learning strategies that can dynamically adjust to different medical question scenarios remains important for Med-VQA and medical Diff-VQA.

## 3. Materials and Methods

### 3.1. Architecture

**Figure 1:**
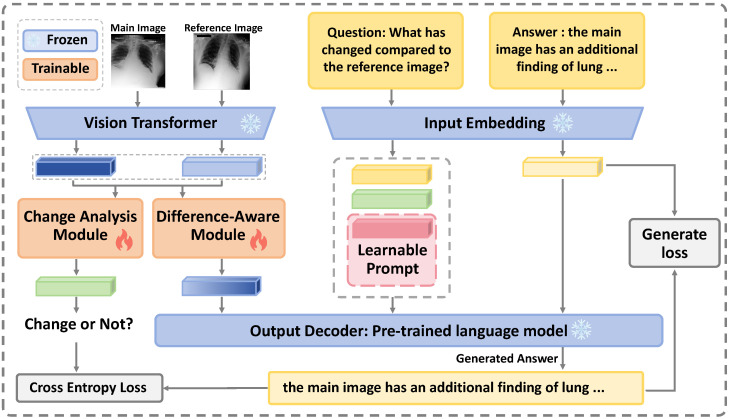
The overall architecture of our proposed method. The Vision Transformer processes both the main and reference images, generating features that are refined by a Change Analysis Module (to detect clinically significant differences) and a Difference-Aware Module (to capture subtle changes). Three types of prompts—a question prompt, a change-aware prompt, and a learnable prompt—are then concatenated and fed into a pretrained language model decoder, which generates the final answer under a generation loss objective. Figure 2 further provides a detailed illustration of the internal operations of the CAM and DAM.

The overall framework is illustrated in Figure 1, which presents the end-to-end workflow from paired image encoding to prompt-conditioned answer generation. Specifically, the model first employs a visual encoder $\Phi_{\mathrm{enc}}$ to separately extract visual features from the current image $I_{\mathrm{main}}$ and the reference image $I_{\mathrm{ref}}$. These features are then fed into two independent modules:

1. **Change Analysis Module** $\Phi_{\mathrm{ca}}$: This module generates a change-vector prompt to indicate whether meaningful differences exist between the two images.
2. **Difference-Aware Module** $\Phi_{\mathrm{diff}}$: This module further processes the extracted visual features to produce a difference-aware representation.

**Figure 2:**
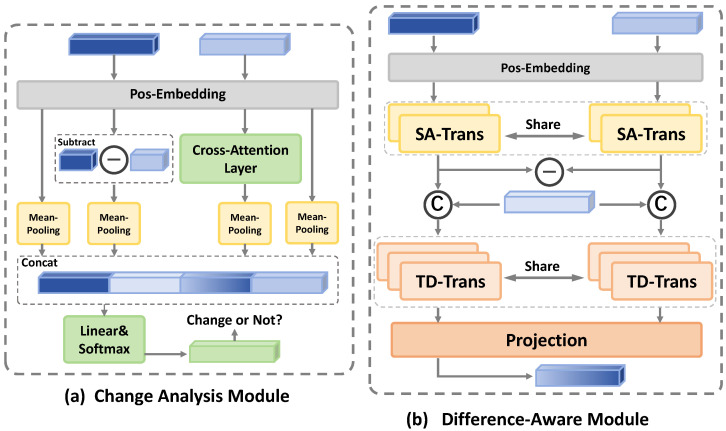
Overall framework combining the Change Analysis Module and the Difference-Aware Module. In (**a**), positional embeddings are added to the main and reference feature maps, which are then differenced and processed by a cross-attention layer; the pooled outputs are concatenated and passed through a linear and softmax layer to produce a binary change prompt. In (**b**), the position-enhanced features undergo spatial alignment via SA-Trans, followed by element-wise subtraction to obtain a raw difference map; both aligned and difference streams are then fed into the TD-Trans for temporal encoding, and a final projection layer yields the difference-aware embedding for answer generation.

Finally, using the GPT-2 language decoder $\Phi_{\mathrm{dec}}$, the model integrates the question prompt $P_{\mathrm{question}}$, the change-vector prompt $P_{\mathrm{changex}}$, an additional learnable prompt $P_{\mathrm{learnable}}$, and the difference-aware representation *z* to generate the final answer. For clarity, Figure 2 further illustrates the internal operations of the CAM and DAM in detail.

The overall workflow of the model can be summarized as follows:

(1) $$\begin{array}{cc} V_{\mathrm{main}},V_{\mathrm{ref}} & =\Phi_{\mathrm{enc}}\left(I_{\mathrm{main}},I_{\mathrm{ref}}\right) \end{array}$$

(2) $$\begin{array}{cc} P_{\mathrm{changex}} & =\Phi_{\mathrm{ca}}\left(V_{\mathrm{main}},V_{\mathrm{ref}}\right) \end{array}$$

(3) $$\begin{array}{cc} z & =\Phi_{\mathrm{diff}}\left(V_{\mathrm{main}},V_{\mathrm{ref}}\right) \end{array}$$

(4) $$\begin{array}{cc} A & =\Phi_{\mathrm{dec}}\left(P_{\mathrm{question}},P_{\mathrm{changex}},P_{\mathrm{learnable}},z\right) \end{array}$$

where $V_{\mathrm{main}}$ and $V_{\mathrm{ref}}$ denote the visual feature representations extracted from $I_{\mathrm{main}}$ and $I_{\mathrm{ref}}$, respectively, and *A* denotes the generated answer.

### 3.2. Core Components

#### 3.2.1. Change Analysis Module (CAM)

Existing Diff-VQA methods often unify the “changed” and “unchanged” cases within a unified generation pipeline, overlooking the inherent imbalance in their descriptive complexity. This often limits the model’s ability to handle “no-change” scenarios concisely while accurately describing subtle disease progression when changes occur. To address this issue, our method explicitly determines whether significant changes exist between the paired images and uses this signal to guide subsequent answer generation.

Given the main and reference image features $V_{\mathrm{main}},V_{\mathrm{ref}}\in R^{L\times d}$ extracted by the visual encoder, we first apply positional encoding:

(5) $$V_{\mathrm{main}}^{\prime }=V_{\mathrm{main}}+E_{\mathrm{pos}},\;V_{\mathrm{ref}}^{\prime }=V_{\mathrm{ref}}+E_{\mathrm{pos}}$$

We compute the element-wise difference between the two embedded features:

(6) $$V_{\mathrm{diff}}=V_{\mathrm{main}}^{\prime }-V_{\mathrm{ref}}^{\prime }$$

To enhance cross-image interaction, we apply a cross-attention layer where the main image serves as the **Query** and the reference image serves as the **Key** and **Value**:

(7) $$V_{\mathrm{ca}}=\mathrm{CrossAtt}\left(Q=V_{\mathrm{main}}^{\prime },\;K=V_{\mathrm{ref}}^{\prime },\;V=V_{\mathrm{ref}}^{\prime }\right)$$

We then perform mean pooling on all intermediate representations:

(8) $$\begin{array}{cc} v_{\mathrm{main}} & =\mathrm{Mean}\left(V_{\mathrm{main}}^{\prime }\right),\;v_{\mathrm{ref}}=\mathrm{Mean}\left(V_{\mathrm{ref}}^{\prime }\right)\\ v_{\mathrm{diff}} & =\mathrm{Mean}\left(V_{\mathrm{diff}}\right),\;v_{\mathrm{ca}}=\mathrm{Mean}\left(V_{\mathrm{ca}}\right) \end{array}$$

These are concatenated to form a unified representation:

(9) $$V_{\mathrm{cat}}=\left[v_{\mathrm{main}};\;v_{\mathrm{ref}};\;v_{\mathrm{diff}};\;v_{\mathrm{ca}}\right]$$

Finally, a linear projection followed by a softmax function produces a binary change prediction:

(10) $$P_{\mathrm{changex}}=\mathrm{Softmax}\left(WV_{\mathrm{cat}}+b\right)$$

where *W* and *b* are learnable parameters. The resulting $P_{\mathrm{changex}}\in R^{2}$ indicates whether a change is predicted to be present and is used as a change-aware prompt for answer generation.

#### 3.2.2. Difference-Aware Module (DAM)

Previous approaches often resort to straightforward subtraction or concatenation of visual features, which may discard subtle yet important difference information and thereby reduce the accuracy of subsequent answer generation. To address this issue, our method introduces an explicit mechanism to capture fine-grained variations between image pairs. By employing a two-stage Transformer architecture, the DAM progressively refines feature representations, providing support for more accurate answer generation.

Following the same preprocessing used in the CAM, we first incorporate positional encodings into the raw visual features extracted by the encoder, resulting in position-enhanced representations $V_{\mathrm{main}}^{\prime },V_{\mathrm{ref}}^{\prime }\in R^{L\times d}$. These features are then independently processed by two spatial-aligned Transformers (S-Trans) to align spatial semantics:

(11) $$V_{\mathrm{main}}^{\prime \prime }=\mathrm{S-Trans}\left(V_{\mathrm{main}}^{\prime }\right),\;V_{\mathrm{ref}}^{\prime \prime }=\mathrm{S-Trans}\left(V_{\mathrm{ref}}^{\prime }\right)$$

After obtaining these aligned features, the DAM explicitly computes the raw difference feature by element-wise subtraction:

(12) $$V_{\mathrm{diff}}^{\mathrm{raw}}=V_{\mathrm{main}}^{\prime \prime }-V_{\mathrm{ref}}^{\prime \prime }.$$

Then, the two pairs $\left\{V_{\mathrm{main}}^{\prime \prime },V_{\mathrm{diff}}^{\mathrm{raw}}\right\}$ and $\left\{V_{\mathrm{ref}}^{\prime \prime },V_{\mathrm{diff}}^{\mathrm{raw}}\right\}$ are separately fed into two weight-shared Transformer modules named temporal difference Transformers (TD-Trans), which output the difference-aware temporal representations:

(13) $$\begin{array}{cc} {\hat{V}}_{\mathrm{main}} & =\mathrm{TD-Trans}(V_{\mathrm{main}}^{\prime \prime },V_{\mathrm{diff}}^{\mathrm{raw}})\\ {\hat{V}}_{\mathrm{ref}} & =\mathrm{TD-Trans}(V_{\mathrm{ref}}^{\prime \prime },V_{\mathrm{diff}}^{\mathrm{raw}}) \end{array}$$

Finally, these outputs are projected into a unified semantic space by a projection function to obtain the final difference representation:

(14) $$V_{\mathrm{diff}}=\mathrm{Projection}\left({\hat{V}}_{\mathrm{main}},{\hat{V}}_{\mathrm{ref}}\right).$$

#### 3.2.3. Multi-Prompt Learning Mechanism (MLM)

Diff-VQA requires context-aware and descriptive answer generation. To better condition the language decoder on the question content and the image change scenario, our method introduces a multi-prompt learning mechanism. Specifically, MLM embeds three distinct prompts into a pretrained GPT-2 language decoder to support answer generation under different scenarios.

MLM consists of the following prompt vectors:

1. **Question Prompt** $\left(P_{\mathrm{question}}\right)$: This prompt encodes the input question and guides the decoder toward the relevant semantic content.
2. **Change-aware Prompt** $\left(P_{\mathrm{changex}}\right)$: Generated by the Existence-of-Change Analysis Module, this prompt indicates whether a difference exists between the two images.
3. **Learnable Prompt** $\left(P_{\mathrm{learnable}}\right)$: Consisting of learnable multi-dimensional vectors that are continuously optimized during training, this prompt provides additional task-adaptive context for answer generation.

These three prompts are first mapped into the same embedding space as the GPT-2 token embeddings and then concatenated to form a multi-prompt input:

(15) $$P_{\mathrm{multi}}=\left\{\;P_{\mathrm{question}},\;P_{\mathrm{changex}},\;P_{\mathrm{learnable}}\right\}.$$

Before decoding, $P_{\mathrm{multi}}$ is concatenated with the token embeddings of the answer sequence and jointly fed into the GPT-2 decoder, while the difference-aware representation $V_{\mathrm{diff}}$ provides additional visual context for generation. The final answer is generated by

(16) $$A=\Phi_{\mathrm{dec}}\left(P_{\mathrm{multi}},\;V_{\mathrm{diff}}\right),$$

where $\Phi_{\mathrm{dec}}$ denotes the GPT-2 language decoder.

### 3.3. Objective Function

The proposed D^2^MNet is trained in a fully supervised two-stage manner. In the first stage, we train the binary classifier in the Change Analysis Module (CAM) to predict whether a change is present between paired images. In the second stage, the output of this classifier is used as a change-aware prompt to guide answer generation.

Stage 1: Change Classification Loss

We formulate the CAM as a two-class classification problem (*change* vs. *no change*) and optimize it using categorical cross-entropy loss:

(17) $$L_{\mathrm{cls}}=-\sum_{i=1}^{N}\left[p_{i}^{\left(\mathrm{cls}\right)}\;\log y_{i}^{\left(\mathrm{cls}\right)}\right],$$

where $y_{i}^{\left(\mathrm{cls}\right)}$ denotes the predicted probability vector produced by the softmax classifier and $p_{i}^{\left(\mathrm{cls}\right)}$ denotes the one-hot vector corresponding to the ground-truth label. We adopt softmax rather than sigmoid because the CAM is formulated as a mutually exclusive two-class prediction problem, where each image pair is labeled as either *change* or *no change*.

Stage 2: Answer Generation Loss

In the second stage, the weights of the CAM classifier are fixed, and its output is used only to generate the change-aware prompt $P_{\mathrm{changex}}$. This design allows the answer generation stage to focus on decoder optimization while preserving the change-aware signal learned in Stage 1.

Let $p_{t}$ denote the predicted probability distribution over the vocabulary at time step *t*, and let $y_{t}^{\left(v\right)}$ be the one-hot vector corresponding to the ground-truth word. The generation loss is defined as

(18) $$L_{\mathrm{gen}}=-\sum_{t=1}^{T}\left[y_{t}^{\left(v\right)}\;\log p_{t}\right],$$

where *T* is the length of the target answer sequence. During this stage, the decoder is conditioned on the multi-prompt input together with the difference-aware representation to generate the final answer.

## 4. Experiments

### 4.1. Datasets and Preprocessing

We evaluate D^2^MNet on the publicly available MIMIC-Diff-VQA dataset [11], which is derived from MIMIC-CXR [34]. The full dataset contains 700,703 question–answer pairs collected from 164,324 medical cases. Its questions are categorized into seven types: abnormality, presence, view, location, type, level, and difference. Since this work focuses on explicitly modeling and describing differences between paired medical images, we conduct experiments on the *difference* subset, which contains 131,563 difference-related question–answer pairs. This setting is also consistent with prior Diff-VQA studies and allows for fair comparison with existing methods. Since the released MIMIC-Diff-VQA benchmark does not provide an explicit binary label indicating whether change is present, we derive the supervision for the CAM automatically from the answer text. In particular, answers expressing the standardized no-change response (e.g., “Nothing has changed.”) are treated as negative samples, while the remaining difference-focused answers are treated as positive samples. This heuristic is practical in the current benchmark because no-change responses are highly regularized in wording, allowing us to construct binary change labels without introducing additional manual annotation.

Following the standard protocol introduced by the dataset authors, we split the selected difference subset into training, validation, and test sets with a ratio of 8:1:1.

During preprocessing, all chest X-ray images are resized to a uniform resolution of 512×512 and normalized. To reduce overfitting, we apply random data augmentation, including brightness, contrast, and saturation adjustment, as well as slight rotation.

For visual feature extraction, we use the ViT-B/32 model from CLIP to obtain grid-level visual features. The text embedding layer is initialized with the pretrained GPT-2 model, which maps input tokens into 768-dimensional embeddings. We adopt GPT-2 as the language decoder to maintain a fair comparison with ReAl, which uses the same decoder setting. This allows the performance differences to be more directly attributed to the proposed CAM, DAM, and MLM components rather than to a stronger generative backbone.

In the CAM, the classification branch contains three Transformer layers. In the DAM, the temporal difference Transformer (TD-Trans) contains two layers, while the spatial-aligned Transformer (SA-Trans) contains three layers. The learnable prompt $P_{\mathrm{learnable}}$ is composed of five trainable tokens. Positional and temporal information are encoded by adding corresponding learnable embedding vectors.

### 4.2. Implementation Details and Evaluation Metrics

All experiments are implemented in PyTorch 1.13 [35]. The pretrained ViT-B/32 visual encoder [36] and the GPT-2 decoder are kept fixed during training. The remaining parameters are optimized using AdamW with an initial learning rate of $1\times 10^{-5}$ and a cosine annealing schedule. Each training run is performed for 20 epochs with a batch size of 16. During inference, beam search with a beam size of 5 is adopted for answer generation. The binary supervision for the CAM is derived from the answer text as described in Section 4.1, rather than provided as an explicit label in the original benchmark. Following the two-stage training strategy described in Section 3.3, we first train the CAM classifier and then fix it to provide the change-aware prompt for the subsequent answer generation stage. All experiments are conducted on a single Linux server equipped with an Intel(R) Xeon(R) Gold 5420+ CPU, one NVIDIA A800 80 GB GPU, and 256 GB memory. Each epoch takes approximately 70 min, resulting in a total training time of about 23.3 h per run. Our model contains 412.58 M parameters in total, of which 198.68 M are trainable.

For evaluation, we use standard natural language generation metrics, including BLEU [37], METEOR [38], ROUGE-L [39], and CIDEr [40]. These metrics are used to assess lexical overlap and semantic similarity between the generated answers and the reference answers.

### 4.3. Comparison with State-of-the-Art Methods

In this section, we compare our proposed method with several representative approaches on the MIMIC-Diff-VQA dataset, including MCCFormers [41], IDCPCL [42], EKAID [11], PLURAL [12], and ReAl [13]. These methods vary in architectural design and learning strategies, ranging from standard image captioning extensions to domain-specific models tailored for medical difference reasoning. A brief overview of each method is provided below. For clarity, Table 1 provides a descriptive summary of the baseline methods compared in this study.

### 4.4. Experimental Results

Table 2 presents a comparison between the proposed D^2^MNet and several representative methods on the MIMIC-Diff-VQA benchmark. All models are evaluated under the same experimental setting, enabling fair comparison across standard natural language generation metrics.

As shown in Table 2, D^2^MNet achieves the best results on six out of seven evaluation metrics and remains highly competitive on BLEU-1. Compared with the strongest baseline, ReAl [13], D^2^MNet improves BLEU-4, METEOR, ROUGE-L, and CIDEr by 2.64%, 1.52%, 1.90%, and 20.68%, respectively. In particular, the substantial gain in CIDEr suggests better overall agreement with the reference answers under benchmark evaluation.

The improvements in BLEU and ROUGE-L also suggest better lexical and semantic alignment between the generated answers and the ground-truth responses. We attribute these gains to the joint effect of the Change Analysis Module (CAM), Difference-Aware Module (DAM), and multi-prompt learning mechanism (MLM). Specifically, the change-aware prompt provides explicit guidance about the image change scenario, while the difference-aware representation supplies complementary cues for answer generation. Together, these components help the decoder generate more appropriate responses to difference-focused medical questions.

**Figure 3:**
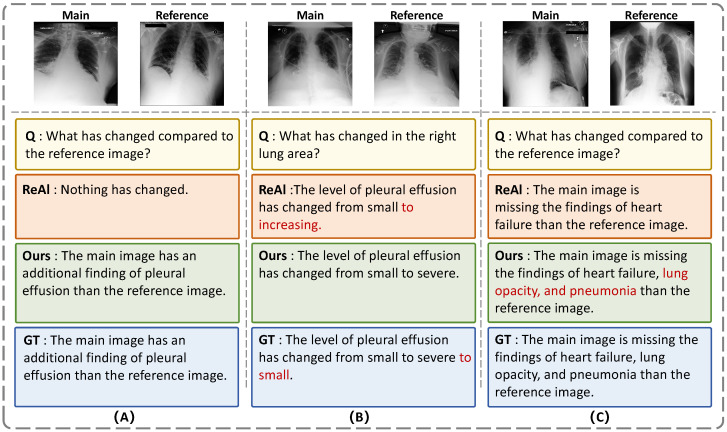
Qualitative comparisons between the ReAl baseline and our model on three representative Diff-VQA cases. In case (**A**), ReAl incorrectly predicts “no change,” whereas our model correctly identifies pleural effusion. In case (**B**), ReAl generates a vague response (“from small to increasing”), while our model provides a more precise description despite inconsistency in the ground truth. In case (**C**), ReAl misses several pathological findings, whereas our model correctly identifies all relevant conditions in agreement with the reference answer. Incorrect or missing predictions are marked in red.

To further illustrate the behavior of the proposed model beyond quantitative results, we provide qualitative comparisons with ReAl in Figure 3. The examples show that D^2^MNet better captures subtle differences in several cases and produces answers that are more consistent with the reference responses.

To better characterize model failures beyond a few qualitative examples, we conducted a pilot manual audit of 20 erroneous predictions and categorized them using the proposed Diff-VQA error taxonomy. The detailed error categorization is summarized in Table 3. The results suggest that change existence errors were relatively rare (5%), indicating that the model was generally effective at recognizing whether a clinically relevant change had occurred. In contrast, severity-related errors were the most frequent, including severity mismatch (25%) and incomplete severity transition descriptions (15%). Additional errors were observed in change direction confusion (20%), finding identification errors (25%), and partial coverage of compound answers (10%). These observations are broadly consistent with the design emphasis of D2MNet: the model appears stronger at detecting and structuring change information than at accurately verbalizing fine-grained severity transitions. This finding suggests that future improvements may benefit from more explicit modeling of severity cues, attribute grounding, and clinically informed decoding strategies.

### 4.5. Ablation Study

**Change Analysis Module:** To verify the effectiveness of the Change Analysis Module (CAM), we conduct an ablation study to examine whether introducing change-aware classification improves performance on Diff-VQA. All other components and training settings are kept identical across the compared variants, so that the observed differences can be attributed to the presence of the CAM and its associated prompt.

As shown in Table 4, the model without the CAM achieves the lowest scores on all evaluation metrics, indicating that explicit change-aware guidance is important for answering difference-focused questions. When the predicted CAM output is introduced as the change-aware prompt, the model consistently improves across all metrics, showing that the additional change signal helps guide answer generation. Using the oracle CAM prompt further yields the best performance on all metrics, which can be viewed as an upper bound when the change classification is fully accurate. The clear trend (Oracle CAM > Predicted CAM > w/o CAM) confirms that decoupling change analysis from answer generation is beneficial. It also suggests that further improvements in change classification accuracy may lead to additional gains in Diff-VQA performance.

**Figure 4:**
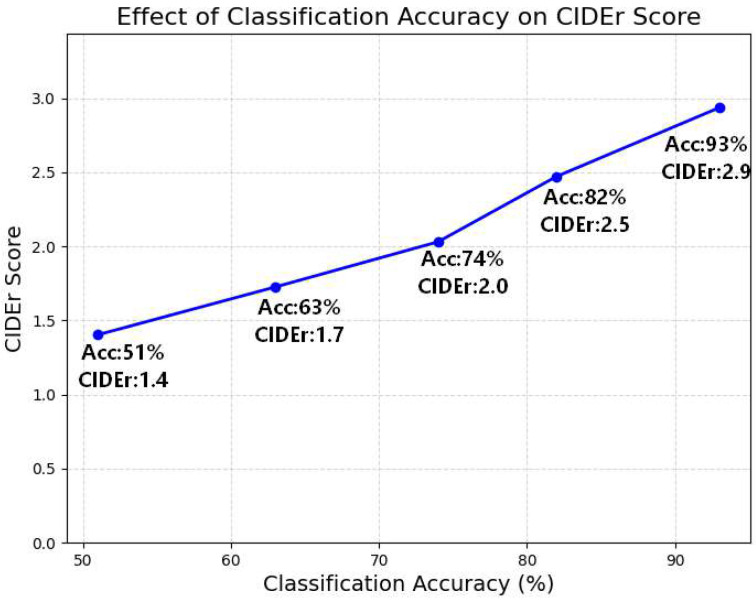
Relationship between classification accuracy and CIDEr score. As the accuracy of the change classification module increases, the CIDEr score of the generated answers improves steadily, indicating a strong positive correlation between accurate change prediction and generation quality.

To further examine the influence of the change classification module, we visualize the relationship between classification accuracy and CIDEr score in Figure 4. The result shows a clear positive correlation: as classification accuracy increases, the CIDEr score also improves, indicating that more accurate change-aware prompts provide more effective guidance for answer generation.

**Difference-Aware Module:** As shown in Table 5, removing the entire Difference-Aware Module (w/o DAM) leads to a substantial drop in performance across all evaluation metrics, indicating the importance of explicitly modeling visual differences between image pairs. When only the Spatial Transformer is retained (Only STr), the performance improves markedly, especially on BLEU-4 and CIDEr, showing that spatial alignment contributes meaningfully to difference representation. The best results are achieved by the full DAM, which combines both spatial and temporal difference modeling. For example, the CIDEr score increases from 2.596 (Only STr) to 2.937 (Full DAM). This result suggests that temporal difference modeling provides complementary information beyond spatial alignment and helps improve answer generation quality. Overall, the comparison validates the effectiveness of the full DAM design for capturing subtle differences between longitudinal medical images.

**Multi-Prompt Learning:** As shown in Table 6, the baseline configuration (G1), which uses only the question prompt ($P_{\mathrm{question}}$), achieves the lowest performance across all metrics, indicating that question information alone is insufficient for effective Diff-VQA answer generation. Adding the change-aware prompt ($P_{\mathrm{changex}}$, G2) improves the results noticeably, with BLEU-4 increasing from 0.266 to 0.340 and CIDEr rising from 0.950 to 1.249. This shows that explicit change-related guidance benefits answer generation. When the learnable prompt ($P_{\mathrm{learnable}}$, G3) is added, the model also improves on several metrics, especially METEOR and ROUGE-L, suggesting that trainable prompts provide complementary task-adaptive context.

The best performance is obtained with the full prompt configuration (G4), which combines $P_{\mathrm{question}}$, $P_{\mathrm{changex}}$, and $P_{\mathrm{learnable}}$. This setting achieves the highest scores on all evaluation metrics, including BLEU-4 of 0.548 and CIDEr of 2.937. The result indicates that the three prompt types are complementary: the question prompt provides semantic guidance, the change-aware prompt conveys explicit change information, and the learnable prompt offers additional adaptive context. Their joint use leads to the most effective answer generation performance on medical Diff-VQA.

## 5. Discussion

### 5.1. Positioning Within Longitudinal Diff-VQA Research

Difference visual question answering in longitudinal radiology can be viewed as a practical research direction for studying difference-focused reasoning and answer generation across time points. In this setting, the objective is not only to recognize findings but also to describe medically relevant changes between paired examinations. Existing Diff-VQA pipelines commonly derive difference representations through direct feature subtraction, concatenation, or residual branches and then rely on a generative decoder to produce free-form answers [11,12,13]. While these paradigms have advanced the field, they often under-specify two aspects that are important for longitudinal difference understanding: (i) the strong asymmetry between “changed” and “unchanged” scenarios in terms of description length and content granularity and (ii) the need for more controllable generation with consistent response behavior. Against this background, D^2^MNet can be viewed as a structured attempt to disentangle *change existence reasoning* from *change description generation*, while improving decoder controllability through prompt-based conditioning.

### 5.2. Why Change-Aware Decoupling Improves Generation Quality

A key observation in longitudinal difference understanding is that “no-change” cases are frequent and can often be answered with concise, standardized statements, whereas “change” cases require richer comparative descriptions. Treating both regimes with a single, uniform generation behavior may therefore lead to verbosity in no-change cases or missing details in change cases. The proposed Change Analysis Module (CAM) provides an explicit change indicator that functions as a lightweight control signal for the decoder. Empirically, the ablation results show that removing the CAM substantially degrades generation performance, while replacing predicted change prompts with oracle prompts yields a consistent upper bound. This trend suggests that improved change recognition can directly translate into better answer generation. Moreover, the monotonic relationship between change classification accuracy and CIDEr suggests that the CAM is not merely an auxiliary classifier, but also a mechanism that improves the alignment between the inferred change scenario and the language generation behavior.

From an optimization perspective, this decoupling also mitigates a form of implicit label ambiguity: without an explicit change signal, the decoder must simultaneously infer *whether* to describe differences and *what* to describe, using only supervision from the target answer string. Introducing the CAM reduces the burden on the generative objective by providing a discrete summary of scenario type, which can stabilize training and reduce over-generation in no-change samples. This interpretation also provides a plausible explanation for the more targeted responses observed in the qualitative examples.

### 5.3. Effectiveness of Fine-Grained Difference Modeling in DAM

The Difference-Aware Module (DAM) is designed to address a second bottleneck: naive differencing can be sensitive to nuisance variations (e.g., view changes, slight positioning differences, and exposure variations) and may suppress subtle difference signals. The DAM’s two-stage design can be interpreted as (i) spatial alignment to reduce irrelevant discrepancies and (ii) temporal-aware differencing to emphasize meaningful changes across time points. The ablation study indicates that spatial alignment alone already brings large gains over direct fusion, while adding temporal differencing further improves the generation metrics. This result supports the view that longitudinal difference reasoning benefits from explicitly modeling evolution patterns rather than relying on a single static difference map.

Importantly, the observed improvements are not limited to metric gains alone. In qualitative comparisons, the DAM appears to help the model maintain sensitivity to subtle differences and reduce omissions of findings reflected in the reference answers. Therefore, modules that enhance difference saliency and reduce noise sensitivity, such as the DAM, are beneficial not only for overall performance but also for robustness under label noise.

### 5.4. Multi-Prompt Learning for Controllable Answer Generation

Recent vision–language systems increasingly rely on prompt-based conditioning to adapt pretrained language decoders to task-specific generation settings [23,24,27,29]. In D^2^MNet, the multi-prompt learning mechanism integrates three complementary signals: (i) the question prompt to preserve the inquiry intent, (ii) the change-aware prompt to control the response regime (changed vs. unchanged), and (iii) learnable soft prompts to capture task-specific priors that are difficult to express with handcrafted templates. The ablation results suggest that these prompts contribute in different ways: the change-aware prompt is particularly helpful for enforcing appropriate response behavior, whereas the learnable prompts improve the contextual adequacy of the generated answers. Their combination yields the best overall performance, indicating that explicit control signals and adaptive soft conditioning are complementary rather than redundant.

Conceptually, this design aligns with the requirements of Diff-VQA answer generation: responses should be appropriate to the question context, concise when no meaningful change is present, and sufficiently descriptive when changes occur. Prompt-based conditioning provides a convenient interface for injecting task information into a frozen decoder without fully fine-tuning the language model. This is particularly useful in medical settings, where annotated data are limited and full fine-tuning may increase the risk of overfitting or unstable generation behavior.

### 5.5. Limitations and Future Directions

Several limitations should be acknowledged. First, the present study primarily evaluates the benchmark-level effectiveness of D^2^MNet rather than its clinical validity. The experiments are conducted on a single benchmark and focus only on the “difference” question subset. While this setting is consistent with current medical Diff-VQA studies and supports fair comparison with prior work, broader validation across question types, imaging institutions, and patient populations is still necessary to assess generalization. Future studies should therefore examine whether the proposed framework remains effective under more diverse imaging distributions, broader longitudinal scenarios, and more heterogeneous clinical question formulations. We also note that the current study does not evaluate D^2^MNet on other imaging modalities such as CT or MRI. Although the overall framework is modality-agnostic at the architectural level, its effectiveness beyond longitudinal chest X-ray data remains unverified. Extending the proposed method to CT or MRI would require dedicated validation and may also require adaptation to modality-specific characteristics, such as volumetric structure, slice-wise consistency, and different reporting conventions.

Second, standard text generation metrics (BLEU, ROUGE-L, METEOR, and CIDEr) are imperfect proxies for answer quality. Although they are useful for benchmark evaluation, they cannot fully capture clinical correctness, factual consistency, or the practical utility of generated responses. More comprehensive evaluation protocols are therefore needed. Complementary evaluations, such as clinically informed entity-level scoring, hallucination analysis, uncertainty-aware calibration, and expert review, would provide a more faithful assessment of the strengths and weaknesses of the proposed model. Although we included a pilot manual error analysis in this revision, broader and more systematic auditing remains necessary. In addition, future work could investigate whether automatic metrics more closely aligned with factual medical consistency can better reflect performance in this task. Another related challenge is that the reference answers themselves may contain occasional inconsistencies, which complicates supervision and highlights an intrinsic difficulty of Diff-VQA benchmarks.

Third, the current pipeline relies on frozen pretrained encoders and decoders, which improves stability but may limit domain adaptation. Lightweight domain-specific adaptation strategies, such as parameter-efficient tuning, distillation, or selective unfreezing, may further improve performance without sacrificing robustness. It would also be valuable to explore whether the proposed change-aware and multi-prompt design can be combined with stronger decoder backbones or more recent medical vision–language foundation models [17,18,19], in order to clarify whether structured change-aware reasoning remains complementary to stronger general multimodal representations.

Finally, several practical challenges remain open. Longitudinal decision-support systems should ideally provide not only a single best answer but also mechanisms for uncertainty estimation and failure detection, especially in borderline cases where changes are subtle or ambiguous. Integrating explicit localization cues (e.g., attention consistency across time points) and incorporating complementary patient context (such as prior reports, clinical notes, or structured metadata) may improve reliability and interpretability. More broadly, future work should include external validation, broader benchmark coverage, and expert-centered evaluation to better understand the practical value and limitations of such methods in real clinical settings. We also note that the current study does not provide a dedicated cross-method quantitative comparison of efficiency, such as parameter count, training time, or GPU cost. Although we have included detailed implementation settings to improve reproducibility, a more systematic efficiency-oriented evaluation would further strengthen the practical assessment of the proposed model.

## 6. Conclusions

This paper proposes a novel framework, D^2^MNet, for difference-aware medical visual question answering tasks. Unlike existing methods, D^2^MNet explicitly decouples the difference description into two independent sub-tasks: change existence analysis and difference-aware feature extraction. Benefiting from the multi-prompt learning strategy and Transformer-based modules, D^2^MNet demonstrates promising potential for capturing subtle yet clinically relevant image differences, thus significantly enhancing the generation quality of medical diagnostic answers. In future work, we will integrate multimodal inputs including medical reports to enrich feature representations, develop lightweight network variants along with pruning and distillation techniques to reduce inference costs, and incorporate few-shot learning and self-supervised pretraining methods to improve generalizability under scarce data conditions.

## Figures and Tables

**Table 1:** Descriptive summary of the baseline methods compared in Section 4.3.

Method	Core Idea	Relevance to This Study
**MCCFormers** [41]	Multi-scale cross-attention Transformer for generic image difference captioning.	Processes bi-temporal features through hierarchical attention layers to capture spatial differences and generate descriptive captions. Represents a general image difference captioning approach rather than a medical Diff-VQA-specific design.
**IDCPCL** [42]	Contrastive pretraining for image difference captioning.	Uses a dual-encoder structure with contrastive learning objectives to enhance differential feature learning, followed by a decoder for caption generation. Emphasizes visual difference learning but does not explicitly model question-guided medical answer generation.
**EKAID** [11]	Knowledge-guided reasoning for medical Diff-VQA.	Introduces expert-defined diagnostic entity graphs and integrates them into a captioning framework to support medical answer generation. Represents one of the earliest task-specific medical Diff-VQA approaches.
**PLURAL** [12]	Pretrained vision–language model for longitudinal chest X-ray Diff-VQA.	Leverages large-scale pretraining on chest X-ray images and associated reports to align visual and textual semantics over time and then fine-tunes on Diff-VQA to describe disease progression or resolution across visits.
**ReAl** [13]	Residual-based difference encoding with semantic alignment.	Uses residual image encoding together with residual feature alignment (RFA) to better capture subtle semantic differences. Serves as a strong recent medical Diff-VQA baseline.

**Table 2:** Comparison of performance between previous SOTA methods and our D^2^MNet on the MIMIC-Diff-VQA dataset. For D^2^MNet, we report the mean and standard deviation over three runs. The best results are shown in **bold**.

Model	BLEU-1	BLEU-2	BLEU-3	BLEU-4	METEOR	ROUGE-L	CIDEr
MCCFormers [41]	0.214	0.190	0.170	0.153	0.319	0.340	0.000
IDCPCL [42]	0.614	0.541	0.474	0.414	0.303	0.582	0.703
EKAID [11]	0.628	0.553	0.491	0.434	0.339	0.577	1.027
PLURAL [12]	0.704	0.633	0.575	0.520	0.381	0.653	1.832
ReAl [13]	**0.710**	0.636	0.580	0.530	0.395	0.736	2.409
**D^2^MNet (Ours)**	0.709 ± 0.006	**0.637 ± 0.005**	**0.591 ± 0.003**	**0.544 ± 0.007**	**0.401 ± 0.003**	**0.750 ± 0.007**	**2.907 ± 0.040**

**Table 3:** Pilot manual error analysis of 20 erroneous predictions using a task-specific Diff-VQA error taxonomy. We manually reviewed 20 erroneous outputs sampled from the test set and categorized them into ten fine-grained error subtypes. The results suggest that change existence errors are relatively rare, whereas severity-related errors are more frequent.

Main Category	Error Subtype	Definition	Count	Percentage
Change existence	False positive change	The model predicts a change when the reference answer indicates no change.	0	0%
	False negative change	The model fails to detect an existing change and predicts no change or misses the major change.	1	5%
Change direction	Addition → Removal confusion	A newly appeared finding is incorrectly described as removed or decreased.	1	5%
	Removal → Addition confusion	A removed or decreased finding is incorrectly described as newly appeared.	3	15%
Finding identification	Incorrect finding substitution	The model identifies the wrong pathology or substitutes one finding for another.	2	10%
	Spurious finding insertion	The model introduces an unsupported finding not reflected in the reference answer.	1	5%
	Omitted finding	The model fails to mention one or more target findings present in the reference answer.	2	10%
Severity/attribute	Severity mismatch	The model predicts an incorrect severity level or severity transition.	5	25%
	Incomplete severity transition	The model mentions a severity-related change but does not fully specify the transition.	3	15%
Composition/coverage	Partial multi-change description	The model captures only part of a compound answer involving multiple simultaneous changes.	2	10%

**Table 4:** Ablation results for the CAM. Three prompt settings are evaluated: (1) **w/o CAM**, where no classification module is used and no change prompt is provided; (2) **Predicted CAM**, where the predicted classification result from the CAM is used as the change prompt; and (3) **Oracle CAM**, where ground-truth change labels are used as an ideal change prompt.

Prompt Type	BLEU-1	BLEU-2	BLEU-3	BLEU-4	METEOR	ROUGE-L	CIDEr
w/o CAM	0.556	0.509	0.453	0.419	0.331	0.586	1.230
Predicted CAM	0.715	0.641	0.591	0.548	0.404	0.754	2.937
Oracle CAM	0.726	0.655	0.603	0.556	0.425	0.760	3.312

**Table 5:** Ablation results for the DAM. We evaluate three model variants: (1) **w/o DAM**, where no spatial or temporal differencing is used and the image features are directly fused; (2) **Only STr**, which uses only the Spatial Transformer for spatial alignment without temporal differencing; and (3) **Full DAM**, which integrates both spatial and temporal difference modeling.

Model Variant	BLEU-1	BLEU-2	BLEU-3	BLEU-4	METEOR	ROUGE-L	CIDEr
w/o DAM	0.486	0.404	0.356	0.308	0.342	0.467	1.107
Only STr	0.673	0.627	0.583	0.526	0.388	0.731	2.596
Full DAM	0.715	0.641	0.591	0.548	0.404	0.754	2.937

**Table 6:** Ablation results for the MLM. Four prompt settings are evaluated: (1) $P_{\mathrm{question}}$ (**G1**), using only the question prompt as a baseline; (2) $P_{\mathrm{question}}+P_{\mathrm{changex}}$ (**G2**), adding the change-aware prompt; (3) $P_{\mathrm{question}}+P_{\mathrm{learnable}}$ (**G3**), adding a learnable prompt embedding; and (4) $P_{\mathrm{question}}+P_{\mathrm{changex}}+P_{\mathrm{learnable}}$ (**G4**), the full prompt configuration.

Prompt Type	BLEU-1	BLEU-2	BLEU-3	BLEU-4	METEOR	ROUGE-L	CIDEr
$P_{\mathrm{question}}$ (G1)	0.402	0.350	0.324	0.266	0.238	0.343	0.950
$P_{\mathrm{question}}$ + $P_{\mathrm{changex}}$ (G2)	0.545	0.459	0.408	0.340	0.256	0.462	1.249
$P_{\mathrm{question}}$ + $P_{\mathrm{learnable}}$ (G3)	0.505	0.473	0.451	0.402	0.329	0.591	1.121
$P_{\mathrm{question}}$ + $P_{\mathrm{changex}}$ + $P_{\mathrm{learnable}}$ (G4)	0.715	0.641	0.591	0.548	0.404	0.754	2.937

## Data Availability

The data presented in this study are available in PhysioNet (MIMIC-CXR Database) at https://physionet.org/content/mimic-cxr/ (accessed on 6 April 2026).
